# Highly-Ductile Magnesium Alloys: Atomistic-Flow Mechanisms and Alloy Designing

**DOI:** 10.3390/ma12121934

**Published:** 2019-06-15

**Authors:** Kotiba Hamad

**Affiliations:** School of Advanced Materials Science & Engineering, Sungkyunkwan University, Suwon 16419, Korea; hamad82@skku.edu

**Keywords:** magnesium alloys, alloy design, mechanical properties, plasticity, multiscale simulation

## Abstract

This special issue, “Highly-Ductile Magnesium Alloys: Atomistic-Flow Mechanisms and Alloy Designing”, was introduced to cover all aspects associated with the improvement of the ductility of magnesium (Mg) materials through controlling the atomistic flow mechanisms in Mg. The structural aspects that can also influence the ductility of polycrystalline Mg alloys, such as grain size and basal texture characteristics, are addressed in this issue. Therefore, we hope that the contributions to the present issue can effectively support the research on the ductility of Mg.

## 1. Introduction

Mg alloys have been extensively researched in recent years. Their low density and high specific strength have compelled engineers to use them as construction materials in vehicles for weight saving applications. However, most applications of Mg alloys have been impeded due to their low ductility and poor formability at room temperature [1,2,3]. The poor ductility in pure Mg is mainly attributed to the difference (by a factor of 100) in the critical resolved shear stress (CRSS) for non-basal slips compared to basal slips. This results in a limited number of slips that can be activated at room temperature (three basal slips), leading to non-uniform plastic deformation, according to von Mises criteria, and low ductility. Due to this difference in CRSS, a strong basal texture, in which the *c*-axis of the crystals is aligned perpendicular to the rolling direction of the sample, evolves in Mg after a primary processing [4,5]. The basal slip systems that have low CRSS cannot accommodate strain along the *c*-axis; rather, the *c*-axis of the crystal rotates along parallel to the loading direction, leading to basal-oriented grains or strong basal texture.

In this regard, for improving the room temperature ductility of Mg, two correlated concepts should be considered: (1) Weakening the strong basal texture that usually forms in Mg after primary processing and (2) increasing the number of slip systems to be activated (more than five) through changing or reducing the CRSS of non-basal systems.

Mg materials with a weak basal texture, in which many grains have the *c*-axis tilted away from the normal direction of the sample, may exhibit high room temperature ductility. This is related to the higher shear stress that can resolve in non-basal planes, resulting in a higher activity of non-basal slips compared to those in strong-basal-textured Mg materials. Wu et al. [6] collected ductility data for various Mg alloys with different microstructural and textural features. A general trend, in which the ductility decreases with increasing grain size and basal texture intensity, was noted from their data. As the effect of the texture on the ductility has been roughly determined, the effect of grain size is related to the role of grain boundaries in enhancing the activity of non-basal slips (prismatic) [7]. Based on these ideas, a careful control of texture and grain size in Mg materials by thermomechanical processing and alloying may lead to improved ductility. These concepts, however, aim at improving the extrinsic ductility by delaying failure rather than improving intrinsic ductility.

Accordingly, to intrinsically improve the ductility, changes in the CRSS and activity of non-basal slip systems are needed. This improvement in ductility was noted in Mg alloys with Y or other rare earth (RE) elements, where the ductility was improved even for coarse-grained and strong-textured alloys. The improved ductility was attributed to the role of solute atoms in changing the CRSS and activity of non-basal slip systems through modifying the atomistic flow mechanisms. In this regard, several concepts related to these mechanisms were introduced and analyzed for various Mg alloys, such as Mg-Y and Mg-RE binary alloys, including: pyramidal-to-basal (PB) transformation [8], cross-slip of pyramidal II dislocations [6], and stacking fault energies [9,10].

In pure Mg, pyramidal dislocations are usually subjected to a thermally activated process in which those dislocations transform into immobile dislocations (a basal-dissociated dislocation structure) that cannot contribute to plastic deformation. The PB transformation, accordingly, leads to low ductility in the pure Mg, which can be aggravated by the difference in CRSS for pyramidal slip and basal slip systems. In addition, the rate of this transformation in pure Mg is much higher than the cross-slip and multiplication rates of pyramidal dislocations [6]. The Y solute in Mg-Y alloys was found to accelerate the pyramidal II-I double cross-slip rather than delaying the PB transformation.

The stacking fault energies in Mg can play an important role in changing the relative activity of various slip systems, and this concept was used to explain the high ductility of Mg-Y alloys. The low first stacking fault energy in Mg-Y alloy can increase the activity of non-basal slip systems, where those faults with low stacking energy can form an external source of pyramidal dislocations (non-basal dislocation), producing the high ductility of this alloy [9].

Based on the above discussion, a deep understanding of the atomistic mechanisms that control the flow behavior of pure Mg, and more importantly, the relationship between them, is essential. In addition, the effect of solute atoms on such mechanisms and the resultant ductility need to be investigated for various Mg alloys, which may be cheaper compared to Mg-Y and compositionally simple as compared to conventional Mg alloys.
